# Enhanced metastatic capacity of breast cancer cells after interaction and hybrid formation with mesenchymal stroma/stem cells (MSC)

**DOI:** 10.1186/s12964-018-0215-4

**Published:** 2018-01-05

**Authors:** Catharina Melzer, Juliane von der Ohe, Ralf Hass

**Affiliations:** Biochemistry and Tumor Biology Laboratory, Department of Obstetrics and Gynecology, Hannover Medical School, Carl-Neuberg-Str. 1, Hannover, D–30625 Germany

**Keywords:** Mesenchymal stem cells, Breast cancer, Tumor microenvironment, Cell fusion, Hybrid cells

## Abstract

**Background:**

Fusion of breast cancer cells with tumor-associated populations of the microenvironment including mesenchymal stroma/stem-like cells (MSC) represents a rare event in cell communication whereby the metastatic capacity of those hybrid cells remains unclear.

**Methods:**

Functional changes were investigated in vitro and in vivo following spontaneous fusion and hybrid cell formation between primary human MSC and human MDA-MB-231 breast cancer cells. Thus, lentiviral eGFP-labeled MSC and breast cancer cells labeled with mcherry resulted in dual-fluorescing hybrid cells after co-culture.

**Results:**

Double FACS sorting and single cell cloning revealed two different aneuploid male hybrid populations (MDA-hyb1 and MDA-hyb2) with different STR profiles, pronounced telomerase activities, and enhanced proliferative capacities as compared to the parental cells. Microarray-based mRNA profiling demonstrated marked regulation of genes involved in epithelial-mesenchymal transition and increased expression of metastasis-associated genes including S100A4. In vivo studies following subcutaneous injection of the breast cancer and the two hybrid populations substantiated the in vitro findings by a significantly elevated tumor growth of the hybrid cells. Moreover, both hybrid populations developed various distant organ metastases in a much shorter period of time than the parental breast cancer cells.

**Conclusion:**

Together, these data demonstrate spontaneous development of new tumor cell populations exhibiting different parental properties after close interaction and subsequent fusion of MSC with breast cancer cells. This formation of tumor hybrids contributes to continuously increasing tumor heterogeneity and elevated metastatic capacities.

**Electronic supplementary material:**

The online version of this article (10.1186/s12964-018-0215-4) contains supplementary material, which is available to authorized users.

## Background

In many cases removal of the tumor mass by surgery and subsequent treatment after early detection of primary tumors such as breast cancer reveal a favorable outcome and good prognosis for the patients. In contrast, spreading of cancer cells in the circulation (blood or lymph vessels) and formation of metastases significantly complicate a therapeutic regimen with predominantly bad prognosis. Properties of cancer cells to liberate from the primary tumor followed by trans-endothelial migration and attachment to distant tissues require a certain plasticity and adaption to a new microenvironment associated with maintenance of self-renewal capacity [1–3]. Consequently, such metastasizing cancer cells acquire new functional properties which simultaneously alter their chemotherapeutic responsiveness as compared to the local cancer cells of the primary tumor. Various mechanisms contribute to functional alterations of cancer cells including release of soluble factors (cytokines, chemokines, enzymes, metabolites), microvesicles and exosomes, induction of a retrodifferentiation program for potential formation of cancer stem-like cells (CSCs), and close interaction with adjacent neighboring cell populations within the heterogeneous tumor tissue such as immune cells, endothelial cells or local tissue-associated mesenchymal stroma/stem cells (MSC).

Although MSC express diverse and multi-functional plasticity according to tissue-specific origin/homing, these heterogenic populations share a variety of common properties including regenerative potential, protection of stemness and tissues, regulation of stem cell homeostasis, support of repair and neovascularization, immune modulation, and differentiation capacity [4–6]. Similar properties are evolved by MSC at tumor sites by interacting with cancer cells [7]. Previous work has demonstrated that different breast and ovarian cancer cells at least transiently acquire new functional properties following interaction with MSC via gap junctional intercellular communication or notch signaling in vitro and in vivo [8, 9]. Moreover, receptor interactions such as the intercellular adhesion molecule ICAM-1 expressed on MSC can directly associate with the transmembrane heterodimeric glycoprotein MUC-1 (CD227, DF3, CA15–3) on the cell surface of breast cancer cells to promote an actin-based cell invasive motility in the tumor cells [10–12]. Furthermore, MSC release a large panel of trophic factors including different interleukins, CCLs, CXCLs, FGFs, EGFs, all forms of TGF-β, and PDGFs [13–17]. In response, breast cancer cells express appropriate receptors such as CCR5 and CXCR4 which promote proliferation and migration signals for further corresponding stimuli like CCL5 and SDF-1 [7, 18–23]. Additionally, interactions of breast cancer cells with populations of perivascular regions including pericytes and MSC contribute to tumor cell dormancy [24]. Such changes of cancer cell properties and function by transient stimulation and interaction with MSC can also be induced permanently e.g. by fusion processes and the generation of new cancer cell hybrid populations.

Cell fusions represent rare processes although important during development of certain tissues such as formation of placenta (fusion of fetal trophoblasts to syncytiotrophoblasts), formation of muscle fibers (repeated fusion of myoblasts to multi-nucleated myocytes), or bone metabolism (fusion of osteoclasts). Moreover, fertilization (fusion of sperm and egg) as a merge of gamete genomes displays a special form of cell fusion [25, 26].

The underlying mechanisms for cell fusion including homo- (fusion of cells from the same population) and heterofusion (hybrid formation of different cell types) are not fully understood yet. However, previous work suggested cell fusion as a rare event which appears to be tightly regulated by involving multiple pathways [27]. Factors identified with fusogenic properties include sequestered or truncated genes originating from viral functions such as the HERV-W retroviral envelope genes that have been domesticated in the mammalian genome and evolving proteins termed syncytin-1 and -2 are predominantly found in syncytiotrophoblasts in placenta tissue [28, 29] but also in tumors including tumors of the breast. Moreover, ASCT-2 (alanine, serine and cysteine selective transporter-2) present on endothelial cells and functioning as a syncytin receptor demonstrated fusion of breast cancer cells with endothelial cells [30]. Alternatively, macrophage fusion and myoblast formation may involve the Dock180 (dedicator of cytokinesis) guanine nucleotide exchange factor in cooperation with ELMO for subsequent Rac1 activation and disproportional regulation of the actin cytoskeleton [31, 32].

Cell fusion can be also detected in existing cancers [33]. Thus, cell fusion can generate aneuploidy, chromosomal instability, and DNA damage, all of which might cause multiple genetic changes and cancer [34, 35].

In the present work, we isolated and characterized different cancer hybrid cell populations following interaction and fusion of MDA-MB-231 breast cancer cells with MSC. These hybrid cells revealed alterations in tumorigenicity, metastatic properties and chemotherapeutic responsiveness when compared to their originating parental populations and to each other.

## Methods

### Cell culture

Human MSC were isolated from umbilical cord explant cultures as reported previously [36] and cultured in αMEM (Sigma Chemie GmbH, Steinheim, Germany) supplemented with 10% of allogeneic human AB-serum (commercially obtained from blood bank, University Campus Lübeck, Germany), 100 U/ml penicillin, 100 μg/ml streptomycin and 2 mM L-glutamine (Sigma). For subculture, MSC were harvested by accutase (Sigma) treatment for 3 min at 37 °C. Although limited by clonal convergence during in vitro expansion [37] neo-natal umbilical cord explanted MSC in low passage numbers (P1 to P4) were used due to their superior capabilities as compared to a heterogeneous local tissue-associated MSC population [5].

Human MDA-MB-231 breast cancer cells were obtained from the ATCC (#HTB-26) and cultivated initially at 1500 cells/cm^2^ in Leibovitz’s L-15-medium (Invitrogen) supplemented with 10% (*v*/v) FCS, 2 mM L-glutamin and 1 mM penicillin/streptomycin.

### Cell line testing and authentication

All cells were tested for mycoplasma by the luminometric MycoAlert Plus mycoplasma detection kit (Lonza Inc., Rockland, ME, USA) according to the manufacturer’s recommendations. Moreover, authentication of MDA-MB-231 and MDA-hybrid populations was performed by short tandem repeat (STR) fragment analysis using the GenomeLab human STR primer set (Beckman Coulter Inc., Fullerton, CA, USA). The STR pattern of MDA-MB-231 cell line demonstrated similar results according to the STR database provided by the ATCC, Manassas, VA, USA.

### Co-culture of MSC with human cancer cell lines and isolation of hybrid cells

For co-culture experiments with MSC populations, MDA-MB-231 breast cancer cells were previously adapted to MSC culture medium. In order to distinguish the different cell types and newly formed hybrid cells within the co-culture, the MSC and the cancer cells have been transduced with a 3rd generation lentiviral SIN vector containing the eGFP and the mcherry gene, respectively, as indicated in previous work [9]. Following 6d of MSC^GFP^/MDA-MB-231^cherry^ co-culture (cell ratio 60:40 at a density of 2000 cells/cm^2^) in MSC medium, the cells were separated by repeated fluorescence-activated cell sorting (FACS) of double-labeled (mcherry and GFP) cells into microtiter plates with one to two hybrid cells/well for subsequent cell cloning. Two resulting hybrid clones of MDA-MB-231 co-culture (MDA-hyb1 and MDA-hyb2) were further analyzed.

### Telomerase assay

The activity of this nuclear enzyme was detected by TRAPeze telomerase detection kit (Millipore, Beverly, MA, USA) in a radioactive assay as previously described [38]. Briefly, homogenates of 10^6^ cells were prepared in CHAPS lysis buffer and combined with the reaction mixture including a [γ-32P] ATP radiolabeled TS primer which has been previously labelled with T4-polynucleotide kinase (NEB, Beverly, MA, USA). Evaluation and adjustment of equal amount of protein was performed using the Bradford assay (Bio-Rad Inc., Richmond, CA, USA). The different cell samples were subjected to PCR amplification according to the manufacturer’s instructions using Taq DNA polymerase (NEB, Beverly, MA, USA). Thereafter, loading dye was added to the amplified DNA and the samples were separated in a 10% non-denaturing polyacrylamide gel electrophoresis. The gel was dried and the radioactive bands were visualized in a PhosphorImager (Storm 820, Amersham Biosciences).

### Transcript analysis by RT-PCR

Total RNA was isolated using RNeasy Mini Kit (Qiagen, Hilden, Germany) according to the manufacturer’s instructions. One μg RNA was reverse transcribed into cDNA using 500 μM of dNTP (R0193), 5 μM Oligo(dT)18 primer (S0132), 5 μM Random Hexan primer (S0142), 1 U RiboLockTM RNase Inhibitor (E00381) and 5 U RevertAidTM M-MuLV Reverse Transcriptase (EP0441) in the supplied reaction buffer (all reagents from Thermo Scientific, Schwerte, Germany). The cDNA reactions were performed for 10 min/25 °C, 1 h/37 °C and stopped at 72 °C for 10 min. As a template 2.5 μl of cDNA was used with primers specific for

- mcherry (sense: 5`-TTC ATG TAC GGC TCC AAG GC-3′; antisense: 5`-CTG CTT GAT CTC GCC CTT CA-3′; amplification product 297 bp)
- eGFP (sense: 5`-CTA TAT CAT GGC CGA CAA GCA GA-3′; antisense: 5`-GGA CTG GGT GCT CAG GTA GTG G-3′; amplification product 165 bp)
- CD73 (sense: 5’-CGC AAC AAT GGC ACA ATT AC-3′; antisense: 5’-CTC GAC ACT TGG TGC AAA GA-3′; amplification product 241 bp) [9];
- CD90 (sense: 5′-GGA CTG AGA TCC CAG AAC CA-3′; antisense: 5’-ACG AAG GCT CTG GTC CAC TA-3′; amplification product 124 bp) [9];
- CD105 (sense: 5′-TGT CTC ACT TCA TGC CTC CAG CT-3′; antisense: 5′-AGG CTG TCC ATG TTG AGG CAG T-3′; amplification product 378 bp) [9]
- MFSD-2A (sense: 5`-CTC CTG GCC ATC ATG CTC TC-3′; antisense: 5`-GGC CAC CAA GAT GAG AAA-3′; amplification product 129 bp)
- syncytin-2 (sense: 5`-AGC AGC CGT AGT CCT TCA AA-3′; antisense: 5`-AGG GGA AGA ACC CAA GAG AA-3′; amplification product 231 bp) [39]
- Ki67 (sense: 5`-TAT CAA AAG GAG CGG GGT CG-3′; antisense: 5`-TTG AGC TTT TCT CAT CAG GGT CA-3′; amplification product 389 bp) [40]
- GAPDH as a control (sense: 5’-ACC ACA GTC CAT GCC ATC AC-3′; antisense: 5’-TCC ACC ACC CTG TTG CTG TA-3′; amplification product 452 bp) [41] (all primers customized by Eurofins, MWG GmbH, Ebersberg, Germany). PCR reactions included 0.2 μM of each primer, 200 μM of dNTP (R0193, Thermo Scientific) and 0.03 U One Taq Hot Start DNA polymerase (New England Biolabs GmbH, Frankfurt am Main, Germany) in the supplied reaction buffer. PCR cycling conditions were performed 30 s at 94 °C, 1 min at 60 °C and 68 °C for 1 min respectively, including an initial 30 s denaturation step at 94 °C and a final 5 min extension step at 68 °C (35 cycles). Aliquots of 25 μl of each RT-PCR product were separated on a 2% agarose gel including the standard GeneRuler 100 bp DNA Ladder (Thermo Scientific) and visualized by GelRedTM (Biotium Inc., Hayward, CA, US) staining.

### Microarray-based mRNA expression analysis (single color mode)

This Microarray study has been performed by use of a refined version of the Whole Human Genome Oligo Microarray 4x44K v2 (AMADID 026652, Agilent Technologies), called ‘026652AsQuintuplicatesOn180k’ (AMADID 054261) developed by the Research Core Unit Transcriptomics of Hannover Medical School. Microarray design was defined at Agilent’s eArray portal using a 4x180k design format for mRNA expression as template. All non-control probes of AMADID 026652 have been selected to be printed five times onto one 180 k Microarray (on-chip quintuplicates). Control probes required for proper Feature Extraction software algorithms were determined and placed automatically by eArray using recommended default settings.

Synthesis of Cy3-labeled cRNA was performed with the ‘Quick Amp Labeling kit, one color’ (#5190–0442, Agilent Technologies) according to the manufacturer’s recommendations. cRNA fragmentation, hybridization and washing steps were carried-out exactly as recommended in the ‘One-Color Microarray-Based Gene Expression Analysis Protocol V5.7’.

Slides were scanned on the Agilent Micro Array Scanner G2565CA (pixel resolution 3 μm, bit depth 20). Data extraction was performed with the ‘Feature Extraction Software V10.7.3.1’ by using the recommended default extraction protocol file ‘GE1_107_Sep09.xml’, except that the minimal number of replicates used to calculate population outliers was set to 5.

Measurements of on-chip replicates were averaged using the geometric mean of processed intensity values of the green channel, ‘gProcessedSignal’ (gPS) to retrieve one resulting value per unique non-control probe. Single Features were excluded from averaging, if they i) were manually flagged, ii) were identified as Outliers by the Feature Extraction Software, iii) lay outside the interval of ‘1.42 x interquartile range‘regarding the normalized gPS distribution of the respective on-chip replicate population, or, iv) showed a coefficient of variation of pixel intensities per Feature that exceeded 0.5.

Averaged gPS values were normalized by global linear scaling. For this, all gPS values of one sample were multiplied by an array-specific scaling factor. This factor was calculated by dividing a ‘reference 75th Percentile value’ (set as 1500 for the whole series) by the 75th Percentile value of the particular Microarray to be scaled (‘Array i’ in the formula shown below). Accordingly, normalized gPS values for all samples (microarray data sets) were calculated by the following formula:

$$ \mathrm{normalized}\ {\mathrm{gPS}}_{\mathrm{Array}\ \mathrm{i}}={\mathrm{gPS}}_{\mathrm{Array}\ \mathrm{i}}\kern0.5em \mathrm{x}\ \left(1500/{75}^{\mathrm{th}}\ {\mathrm{Percentile}}_{\mathrm{Array}\ \mathrm{i}}\right). $$

A lower intensity threshold (surrogate value) was defined based on intensity distribution of negative control features. This value was fixed at 10 normalized gPS units. All of those measurements that fell below this intensity cutoff were substituted by the respective surrogate value of 10. Gene expression levels of more than 2-fold difference were compared between parental MSC and hybrid cells as well as between corresponding cancer and hybrid cells and the data file are included as Additional file 1: Figure S9 and stored with the accession no. #GSE100551 at the NCBI-GEO database.

### Cell cycle analysis

For cell cycle analysis in the MDA-hybrid cells and the parental cell lines, 10^5^ cells were fixed in 70% (*v*/v) ice-cold ethanol at 4 °C for 24 h. Thereafter, the fixed cells were stained with 500 μL of DNAse-free RNase (200 U/mL) and 500 μL of propidium iodide (PI) buffer (10 mL PBS + 100 μL Triton-X-100 + 500 μL 0.5 mg/mL propidium iodide in water) in the dark at room temperature for 30 min. The stained cells were analyzed for DNA content in a FACSCalibur (BD Biosciences) using FlowJo software.

### siRNA knock-down

A reverse transfection was applied for knock-down according to the manufacturer’s protocol (Dharmacon, GE Healthcare) using S100A4 small-interfering RNA (siRNA). The breast cancer cell line MDA-MB-231 was transfected with 25 nM S100A4 siRNA (siGENOME Human S100A4 siRNA SMARTpool, cat. #M-004792-01) or non-targeting siRNA (siGENOME Non-Targeting siRNA #3, cat. #D-001210-03, Dharmacon, GE Healthcare) using DharmaFECT1 transfection reagent (cat. #T-2001-02, Dharmacon, GE Healthcare) diluted 1:2000 in transfection medium (antibiotic-free medium) for 24 h. After 24 h transfection, the cells were washed and cultured in normal growth medium. Examination of transfection efficiency was analyzed by flow cytometry following transfection of MDA-MB-231 cells with 25 nM of the green fluorescing siGLOGreen (cat. #D-001630-01, Dharmacon) for 24 h.

For quantification of fusion cells, lentiviral transduced MDA-MB-231^cherry^ cells were transfected for 24 h in 24-well plates with a cell density of 3500 cells/cm^2^ in triplicate. After transfection with siRNA S100A4 and non-targeting siRNA, respectively, the same amount of lentiviral transduced MSC^GFP^ was added and co-cultures were initiated for up to 72 h. At indicated time points, each well was screened for dual fluorescent cells (double positive for GFP and cherry) with green and red fluorescence filters, and a FITC/TRIC fluorescence dual band filter using a fluorescence microscope (Olympus IX50). Following quantification of hybrid cells in the wells, all co-cultures were trypsinized and counted in a hemocytometer to calculate the percentage of dual fluorescent cells.

### In vivo experiments

Animal research using NOD/scid mice was carried out by following the internationally recognized guidelines on animal welfare and has been approved by the institutional licensing committee (Nieders. Landesamt für Verbraucherschutz und Lebensmittelsicherheit) ref. # 33.19–42,502–04-15/1992 on Dec. 18th, 2015.

About 2 × 10^6^ GFP-labeled MDA-MB-231 cells and in separate groups, 2 × 10^6^ MDA-hyb1 and MDA-hyb2 cells were injected subcutaneously into 5 animals of 5 to 6 weeks old female NOD/scid mice, respectively. After 18d post injection, all 15 mice had developed subcutaneous tumors and the animals were sacrificed by CO_2_ anesthesia and cervical dislocation. For a comparable tumor size, additional 4 NOD/scid mice were injected with 2 × 10^6^ GFP-labeled MDA-MB-231 cells and the mice were sacrificed after 34d post injection when the tumor size had reached the range of MDA-hyb1 tumors after 18d post injection.

In vivo treatment experiments were carried out after subcutaneous injection of MDA-MB-231^GFP^ cells and MDA-hyb1 cells into 6 animals, respectively. Four days after tumor cells injection oral therapy (200 μL) was started twice a week with 25 mg/kg taxol in 3 mice and a solvent control (25% ethanol in PBS (*v*/v)) in the other 3 mice of both tumor cell populations. All gavage treatments were carried out using sterile straight 18-gauge, 30 mm plastic feeding tubes (Instech Laboratories Inc., Plymouth, PA, USA). Following tumor cell injection, all 12 mice had developed subcutaneous tumors and the animals were sacrificed by cervical dislocation.

Primary tumor tissues were dissected under UV light and weighted. Organs were also dissected from the mice and thin sections were analyzed by fluorescence microscopy for presence and accumulation of metastatic cells.

### Cytotoxicity measurements of chemotherapeutic-treated cell cultures by fluoroskan assay

The proliferative capacities of MDA-MB-231^GFP^, MDA-hyb1, and MDA-hyb2 cells were compared by fluorescence measurement using the fluoroskan assay as previously described [42, 43]. Briefly, 3000 cells/well were seeded with standard culture medium (100 μL/well) in flat bottom 96-well plates (Nunc/ThermoFischer Scientific, Roskilde, Denmark) and incubated overnight to allow attachment. Thereafter, 100 μL of culture medium was added to the cells as a control and in further wells 100 μL of culture medium containing different chemotherapeutics were added to the cells. Following incubation for up to 72 h, the medium was removed and the cells were lysed with 5% (*w*/*v*) SDS. Afterwards, the fluorescence intensities of mcherry and eGFP in the cell homogenate which corresponded to the appropriate cell number of cancer cells were measured at excitation 485 nm / emission 520 nm (for MDA-MB-231 and MDA-hyb2) or at excitation 584 nm / emission 612 nm (for MDA-hyb1) using the Fluoroskan Ascent Fl (ThermoFisher Scientific).

## Results

Co-culture of different human MSC^GFP^ with mcherry-labeled breast cancer cells was accompanied by formation of hybrid cells via spontaneous cell fusions. Two resulting hybrid clones of MSC^GFP^/MDA-MB-231^cherry^ co-culture (MDA-hyb1 and MDA-hyb2) were selected by subsequent two step cell sorting (Additional file 2: Figure S1). One of these clones lost the GFP gene during the selection process (MDA-hyb1) while the other clone remained double-labeled (MDA-hyb2).

**Fig. 1:**
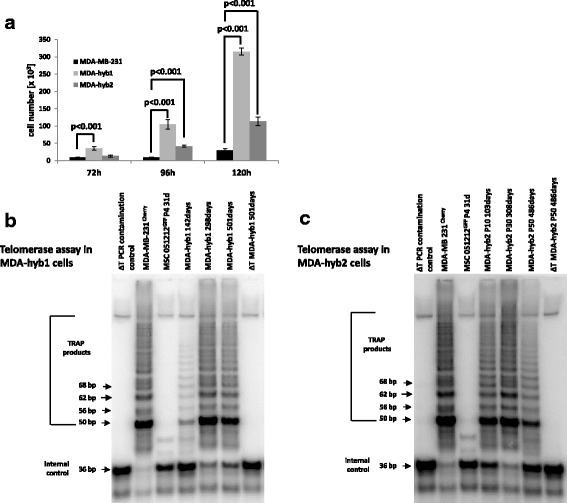
Elevated proliferative potential and sustained telomerase activity in MDA-hyb1 and MDA-hyb2 cells. **a** Proliferative capacity of the different breast cancer populations was tested by cell counting of initially seeded 2.5 × 10^3^ cells in 24-well plates after 72 h to 120 h, respectively. All tumor cell cultures progressively increased in cell number over time. A significantly elevated 10-fold enhanced proliferation for MDA-hyb1 and a 4-fold increased cell growth for MDA-hyb2 hybrid cell population were observed as compared to their wildtype MDA-MB-231 tumor counterpart. Data represent the mean + s.d. of 3 independent experiments. **b** and **c** Telomerase activity was measured in hybrid populations at different ages including MDA-hyb1 after 142d, 298d, or 501d in culture, respectively and MDA-hyb2 in passage 10 (P10), P30, or P50, respectively, in comparison to the parental MSC051212^GFP^ P4 and MDA-MB-231^cherry^ cells. A primer/dimer control and a heat-inactivated telomerase in MSC samples served as an appropriate negative control in the assays

Proliferative capacity of the three different breast cancer populations was tested by cell counting of initially seeded 2.5 × 10^3^ cells in 24-well plates after 72 h to 120 h, respectively (Fig.1a). All tumor cell cultures progressively increased in cell number over time. A significantly elevated hybrid cell population was observed with a 10-fold enhanced proliferation for MDA-hyb1 and a 4-fold increased cell growth for MDA-hyb2 as compared to their wildtype MDA-MB-231 tumor counterpart (Fig.1a).

A sustained telomerase activity substantiated a persistent cell growth of the fused hybrid cell populations as detected for MDA-hyb1 (Fig.1b) and MDA-hyb2 (Fig.1c) in comparison to prominent telomere adducts in the immortalized parental MDA-MB-231 cell line and a weak telomerase activity in the parental MSC (Fig.1b, c).

**Fig. 2:**
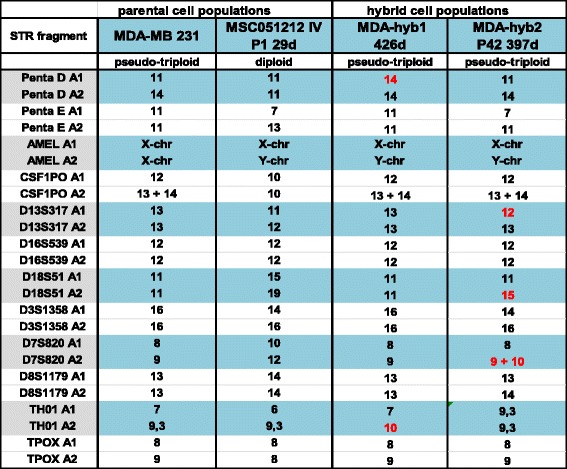
STR fragment analysis of MDA-hyb1 and MDA-hyb2 cells. Characterization by short tandem repeat (STR) fragment analysis was performed for the parental breast cancer MDA-MB-231 cells and MSC051212 in comparison to their spontaneous fusion products MDA-hyb1 and MDA-hyb2. Changes in the STR fragment pattern of the hybrid populations compared to the corresponding parental counterparts are labeled in red

Characterization of the different populations by STR fragment analysis confirmed the known patterns of the parental MDA-MB-231 cell line and revealed some significant differences in the corresponding MDA-hyb1 and MDA-hyb2 cells after spontaneous fusion with individual MSC populations during co-culture (Fig.2). To provide parental populations of different gender, MSC from a male donor were selected for co-culture with the female breast cancer cells and chromosomal rearrangements in the hybrid cells revealed an acquisition of the MSC-derived XY configuration in the analyzed hybrid populations (Fig.2). Karyotype analysis confirmed the male sex of MDA-hyb1 whereby some MDA-hyb2 cells lost the Y-chromosome. Both hybrid populations displayed a pseudo-triploid karyotype like in the parental MDA-MB-231 cells (Additional file 3: Figure S2). Supportive evidence was obtained from steady state cell cycle analysis. DNA fluorescence measurements of normal diploid chromosomes in G1 phase of MSC was enhanced by nearly 50% in MDA-MB-231 cells with a shift in G1 peak and the whole cell cycle demonstrating aneuploidy (Additional file 4: Figure S3). Similar results were obtained for MDA-hyb1 and MDA-hyb2 cells with a pseudo-triploid G1 peak as observed for MDA-MB-231 cells (Additional file 4: Figure S3). Moreover, MDA-hyb1 and MDA-hyb2 cells exhibited a markedly elevated S and G2/M phase, respectively, in contrast to the parental MSC or MDA-MB-231 cells further substantiating a more active proliferation of these hybrid cells (Additional file 4: Figure S3). Interestingly, Ki67 staining (Additional file 5: Figure S4 upper panel) and overall expression of Ki67 (Additional file 5: Figure S4 lower panel) remained at reduced or unaltered levels in MDA-hyb1 and MDA-hyb2 cells suggesting that only parts of these populations exhibit high proliferative capacity.

Together with the appearance of various different STR fragments as compared to the parental cells, these findings demonstrated the generation of heterogeneously proliferating and completely altered hybrid breast cancer cell populations.

**Fig. 3:**
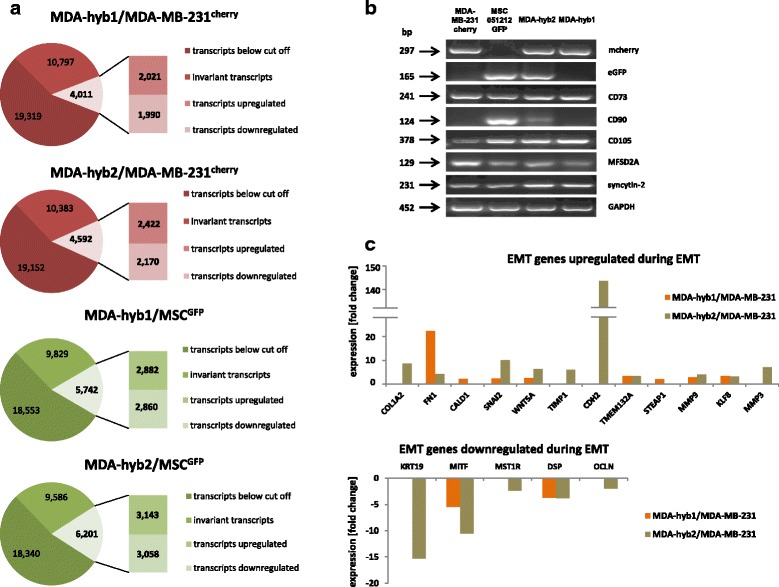
Microarray and PCR analysis of the two hybrid populations in comparison to the parental cell lines. **a** Quantification of genes with changed expression levels (up-regulation = numbers in upper rectangular; down-regulation = numbers in lower rectangular) in MDA-hyb1 (upper panels, red and green) and MDA-hyb2 cells (lower panels, red and green) was performed following microarray analysis with comparison to the parental MDA-MB-231^cherry^ cells (red panels) and to the parental MSC051212^GFP^ (green panels) respectively. Changes in expression levels of more than 2-fold were considered as up- or down-regulated. **b** PCR analysis was performed with mcherry, eGFP, fusion-associated factors syncytin-2 and MFSD-2A, and MSC stem-like markers CD73, CD90, and CD105 expression in the parental MDA-MB-231 and MSC populations as compared to the two hybrid populations. Unaltered GAPDH transcripts served as loading control. **c** Comparison of EMT-related gene expression in MDA-hyb1 relative to MDA-MB-231 (orange-colored bars) and in MDA-hyb2 relative to the parental MDA-MB-231 cells (kaki-colored bars). A variety of genes are up-regulated during EMT (upper panel) in parallel to a down-modulation of other genes (lower panel) to enable development of a mesenchymal phenotype

More detailed analysis of these newly-formed hybrid cell types was performed by RNA microarray profiling (Additional file 1: Figure S9). MDA-hyb1 cells revealed a different expression of 4011 genes compared to MDA-MB-231 cells and 5742 altered expression levels compared to the corresponding MSC (Fig.3a). An even more pronounced alteration was observed in MDA-hyb2 cells with 4592 altered gene expressions compared to the parental MDA-MB-231 cells and 6201 varied mRNA levels compared to the parental MSC, whereby about half of them were up- or down-modulated (Fig.3a).

Exclusive expression of mcherry was detectable in MDA-MB-231^cherry^ and eGFP in MSC^GFP^ cells (Fig.3b). Analysis of the two hybrid population revealed both fluorescence transcripts in MDA-hyb2 cells whereas MDA-hyb1 cells only demonstrated mcherry. The MSC-characteristic stemness markers CD73 and CD90 were differentially expressed in all cell types as evaluated by PCR and RNA microarray analysis, respectively (Fig.3b, Additional file 6: Figure S5). Thus, mRNA levels of the GPI-anchored ecto-5′-nucleotidase (CD73) were significantly enhanced in MDA-hyb1 and even further elevated in MDA-hyb2 cells (Fig.3b, Additional file 6: Figure S5). CD90 was undetectable in the MDA-MB-231 and MDA-hyb1 cells. In contrast, low mRNA levels of CD90 were expressed by MDA-hyb2 when compared to MSC controls (Fig.3b, Additional file 6: Figure S5). Regarding potential fusion properties, all four cell populations expressed transcripts of some fusogenic factors including MFSD-2A and syncytin-2 whereby MFSD-2A has been described as the receptor for syncytin-2 during trophoblast fusion [44].

Further marker, such as CD29 exhibited higher levels in the hybrid cells as compared to MDA-MB-231. In contrast, the hyaluronan receptor CD44 like CD146 and CD166 displayed lower mRNA transcripts in the hybrids compared to both parental populations (Additional file 6: Figure S5). CD105 expression remained nearly unchanged in MDA-MB-231 and the two hybrid populations although significantly lower as compared to MSC (Additional file 6: Figure S5). Together, these findings further characterized the two hybrid populations as completely different cell types whereby MDA-hyb1 exhibited more similarities to MDA-MB-231 cells in contrast to MDA-hyb2 cells expressing certain properties associated with MSC.

Liberation from the primary tumor site, followed by trans-epithelial and trans-endothelial migration as circulating tumor cells in blood or lymphatic vessels represents a necessity for breast cancer cells to form disseminated distant metastasis [45]. This development is predominantly associated with an epithelial-mesenchymal transition (EMT) of the cancer cells although EMT is not absolutely required for tumor dissemination but dispensable in certain kinds of metastasizing tumors [46, 47]. A variety of genes are up-regulated during EMT in parallel to a down-modulation of other genes to enable development of a mesenchymal phenotype. Expression analysis in MDA-hyb1 and even more pronounced in MDA-hyb2 revealed appropriate up-regulation of EMT-associated genes including SNAI2, collagen and fibronectin (Fig. 3c). Moreover, expression of N-cadherin was increased along with matrix metalloproteinases MMP3 and MMP9 (Fig. 3c). In addition, down-regulation was detected for certain genes such as cytokeratin 19, desmoplakin, and some basic helix-loop-helix-associated transcription factors such as MITF (Fig. 3d). These data suggested potential metastatic properties of MDA-hyb1 and MDA-hyb2 cells.

**Fig. 4:**
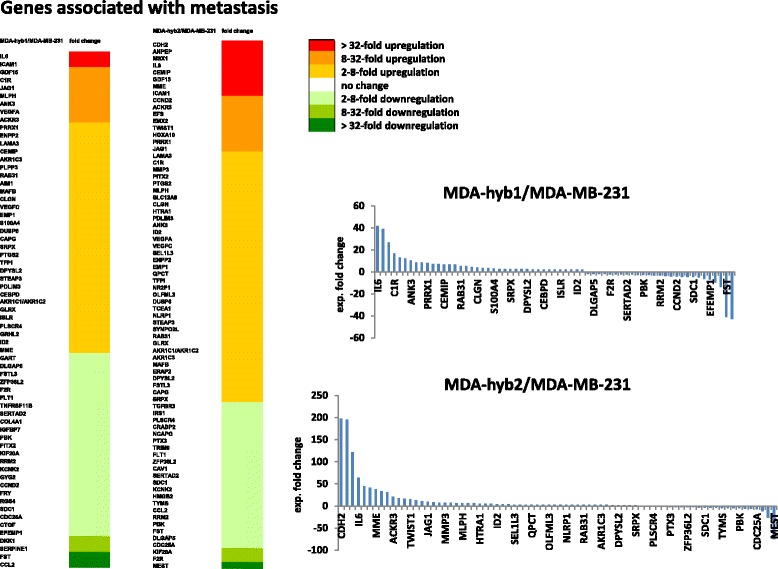
Genes associated with metastasis in MDA-hyb1 and MDA-hyb2 cells. Transcript profile of metastasis-associated gene expression in MDA-hyb1 versus MDA-MB-231 (heat map, left panel) and quantification of selected genes (upper right panel). A similar comparison of metastasis-associated gene expression was performed in MDA-hyb2 versus the parental MDA-MB-231 cells (heat map, right panel) with quantification of selected genes (lower right panel)

Supportive evidence for this suggestion was obtained by the expression profile of metastasis-related gene expression in the two hybrid populations versus the parental MDA-MB-231 breast cancer cell line which revealed a significant up-regulation of a majority of metastasis genes in MDA-hyb1 compared to MDA-MB-231 and likewise in MDA-hyb2 compared to MDA-MB-231 cells (Fig. 4). Together, these findings suggested an enhanced metastatic capacity of the hybrid populations over the parental MDA-MB-231 cells. Indeed, evaluation and comparison of certain disease- and function-associated gene clusters in MDA-hyb1 and MDA-hyb2 hybrid cells as compared to the parental MDA-MB-231 and MSC051212 revealed a significantly elevated expression level of gene clusters accompanied with certain malignancies including different kinds of cancer, migratory capacity, and formation of metastasis (Additional file 7: Figure S6).

**Fig. 5:**
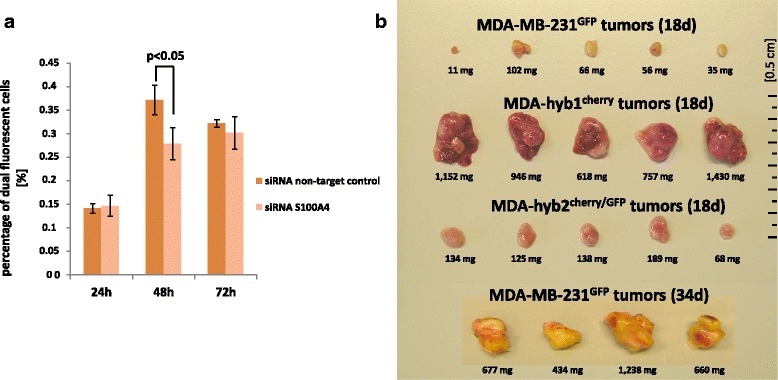
Effects of S100A4 knock-down in hybrid cell formation and in vivo tumor development of hybrid cells. **a** Efficiency and significance of hybrid cell formation by MDA-MB-231^cherry^ and MSC^GFP^ fusion was examined and quantified following siRNA targeted down-modulation of S100A4 versus a non-targeting siRNA. The relative amount of hybrid cells was calculated by the mean ± s.d. from 3 different co-cultures and significance was calculated by student’s-t-test. **b** In vivo tumor development was tested after simultaneous subcutaneous injection of 2 × 10^6^ cells of MDA-MB-231^GFP^, MDA-hyb1, or MDA-hyb2 cells into 5 NODscid mice respectively. The fifteen mice were euthanized after 18d and dissected tumors were compared for tumor size. In addition, 2 × 10^6^ cells of MDA-MB-231^GFP^ cells were injected subcutaneously into 4 NODscid mice. to achieve a comparable tumor size like MDA-hyb1 cells after 34d

Although MDA-hyb1 cells demonstrated increased transcripts associated with metastatic capacity, MDA-hyb2 cells revealed further enhanced expression of EMT genes and more metastasis-associated mRNAs. A direct comparison of all elevated metastasis genes in the microarray data of MDA-hyb1 with those of the MDA-hyb2 expression profile revealed 31 identical and 7 different genes in MDA-hyb1, among them S100A4 which encodes a member of the S100 calcium-binding protein family supporting tumorigenic proliferation by stimulating angiogenesis and by promoting metastases [48]. Calcium-binding induces a conformational change in S100A4 to enable interaction with other proteins such as p53, liprin-β1 or factors involved in cell migration including actins, non-muscle myosin heavy chain IIA, and tropomyosin [49]. Based upon these functionalities, the role of S100A4 in MDA-MB-231 cells and hybrid formation with MSC was elucidated by siRNA knockdown experiments. S100A4 targeting in MDA-MB-231 cells with a transfection efficiency of 92.5% (Additional file 8: Figure S7) was associated with a significantly reduced formation of hybrid cells following co-culture with MSC as compared to a non-targeting siRNA after 48 h (Fig. 5a). These findings suggested that blocking S100A4 signaling reduces the formation of high proliferative active breast cancer hybrid cells also carrying metastatic properties. Supportive evidence is presented by recent work demonstrating reduced migration and metastases of breast cancer cells following down-modulation of S100A4 [50].

**Fig. 6:**
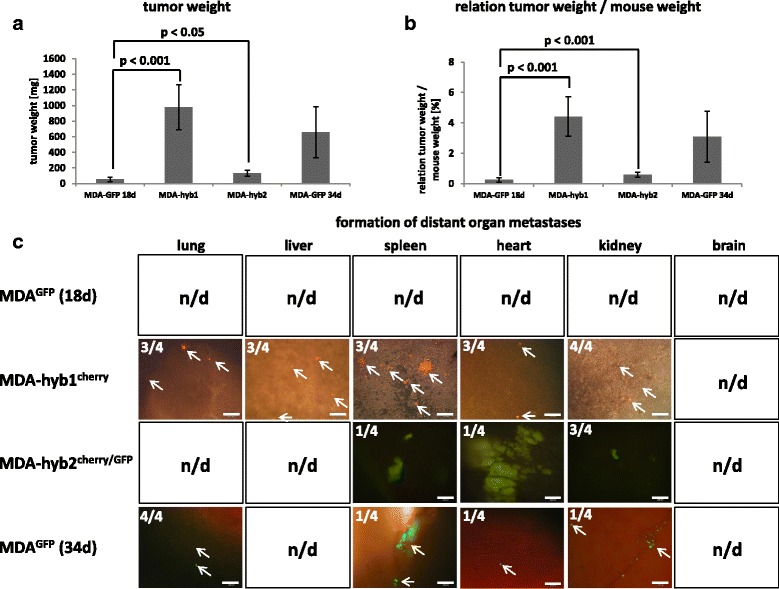
Tumor and metastases formation of MDA-hyb1 and MDA-hyb2 cells. **a** Quantification of the tumor weight was performed after dissection of the solid subcutaneous primary tumors only (disregard of metastatic tumor tissue) derived from the parental MDA-MB-231^GFP^ cells (MDA-GFP) in comparison to mcherry-labeled MDA-hyb1 and mcherry/GFP-labeled MDA-hyb2 populations. Data were obtained by the mean ± s.d. from 5 different tumors of each cell type after 18d. Moreover, additional 4 different MDA-GFP tumors were analysed after 34d. Significance was calculated by student’s-t-test. **b** Quantification of the relationship of tumor weight / mouse weight was calculated with tumors of the parental MDA-MB-231^GFP^ cells (MDA-GFP) in comparison to mcherry-labeled MDA-hyb1 and mcherry/GFP-labeled MDA-hyb2 populations. Data were obtained from 5 different tumors of each cell type after 18d. Moreover, additional 4 different MDA-GFP tumors were analysed after 34d. Significance was calculated by student’s-t-test. **c** Organs from four mice from each injected tumor cell population were dissected after euthanization and evaluated by fluorescence microscopy whereby appropriate fluorescence should indicate formation of distant metastases. Appearance of organ metastases from all of these 16 mice was quantified and exemplary pictures from tissue thin section phase contrast / fluorescence microscopy of organ metastases were documented. n.d. = not detectable. Bars represent 100 μm

To substantiate potentially enhanced tumorigenicity of MDA-hybrid cells and corresponding knowledge in an in vivo system, testing of metastatic capacity was performed after subcutaneous injection of 2 × 10^6^ MDA-MB-231^GFP^, MDA-hyb1, and MDA-hyb2 cells into NODscid mice, respectively. Following simultaneous injection, the hybrid populations developed a substantially increased tumor growth as compared to the parental MDA-MB-231 cells after 18d post injection (Fig. 5b). Using the parental MDA-MB-231-generated tumors (*n* = 5) as a reference, tumor weight was significantly elevated by 18.1-fold in MDA-hyb1 (n = 5) and by 2.4-fold in MDA-hyb2 (n = 5) tumors following 18d after tumor cell injection (Fig. 6a). Similarly, the relationship of tumor weight / mouse weight of 0.25 ± 0.13 in MDA-MB-231^GFP^ mice increased by 17.7-fold to 4.42 ± 1.31 in MDA-hyb1 and by 2.4-fold to 0.60 ± 0.16 in MDA-hyb2 (Fig. 6b). To reach an approximately equivalent tumor size like MDA-hyb1-induced tumors, the parental MDA-MB-231 required 34d after tumor cell injection (Fig. 5b).

Four out of the five mice from each tumor cell population were randomly chosen and screened for potential metastases. Formation of distant organ metastases was analyzed by removal of organs from all 12 mice followed by tissue thin section phase contrast / fluorescence microscopy. None of the organs of previously MDA-MB-231^GFP^–injected cells displayed any detectable GFP fluorescence in the 4 mice after 18d. In contrast, 3 out of 4 mice exhibited metastases to the lung, liver, spleen, and heart, and all (4/4) to the kidney in MDA-hyb1-induced tumors (Fig. 6c). Likewise, organ metastases of MDA-hyb2-derived tumors were detectable in spleen (1/4), heart (1/4), and kidney (3/4). An appropriate size of MDA-MB-231^GFP^-induced tumors after 34d of xeno-transplantation (Fig. 5b) revealed distant organ metastases in the lung (4/4), spleen (1/4), heart (1/4), and kidney (1/4) whereby GFP-positive cells in the liver remained undetectable (Fig. 6c).

While MDA-MB-231 cells as triple negative breast cancer cell line (negative for estrogen, progesterone, and Her2/neu receptors) are long time known for metastatic tumor growth [51] this capability is significantly enhanced and much more accelerated in the MDA-hyb1 and MDA-hyb2 populations.

Taken together, these data demonstrated an expression profile with elevated EMT- and metastasis-associated genes in MDA-hybrid populations which was functionally substantiated in vivo by significantly enhanced tumor growth and much faster formation of multiple organ metastases.

**Fig. 7:**
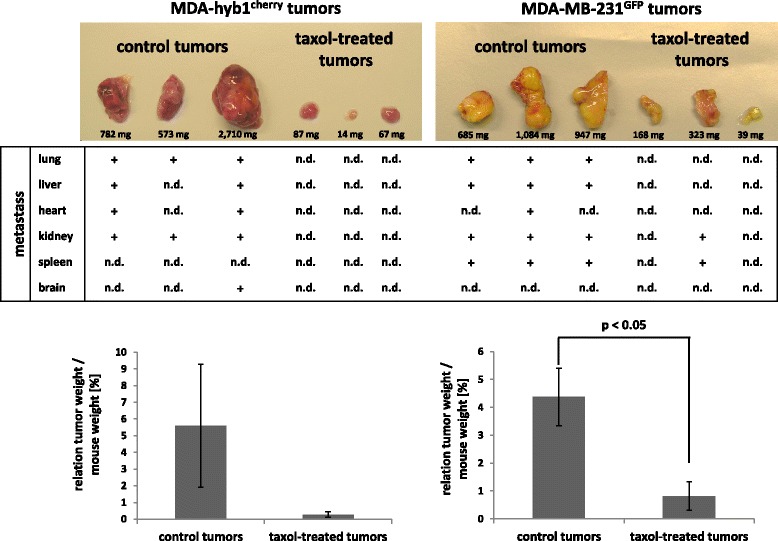
In vivo chemotherapeutic responsiveness of MDA-hyb1 cells. In vivo chemotherapeutic response to 25 mg/kg taxol was tested after subcutaneous injection of 2 × 10^6^ MDA-MB-231^GFP^ cells or 2 × 10^6^ MDA-hyb1^cherry^ cells into 6 NODscid mice, respectively (3 mice for control and 3 mice for taxol-treatment). Based on an average weight of 20 g/mouse gavage was performed with 0.5 mg/200 μL taxol twice weekly and 200 μL solvent in control mice starting 4d after tumor cell application. The twelve mice were euthanized after 22d (MDA-Hyb1^cherry^) and 26d (MDA-MB-231^GFP^) and dissected tumors were quantified for tumor size and analyzed for distant organ metastases by fluorescence microscopy (n.d. = not detectable)

Of interest, MDA-hyb1 and MDA-hyb2 cells demonstrated increased chemotherapeutic sensitivity to a variety of compounds including taxol, cisplatin, methotrexate (MTX), epirubicin, and foretinib after in vitro incubation for 24 h up to 72 h when compared to the parental MDA-MB-231 cells (Additional file 9: Figure S8). To substantiate these findings in vivo, taxol was chosen to evaluate the responsiveness of MDA-hyb1-induced and parental MDA-MB-231-generated NODscid mice tumors (Fig. 7). Whereas MDA-hyb1-mediated tumors developed heterogeneously with different types of metastases, an increased sensitivity to taxol was detectable and this treatment was also associated with no detectable metastases. Parental MDA-MB-231-induced tumors also developed various organ metastases and displayed a certain taxol-sensitivity, however, some organ metastases in kidney (1/3) and spleen (1/3) remained detectable (Fig. 7).

## Discussion

Invasive tumor growth and development of solid tumors is associated with an inflammatory environment, acidic pH, hypoxia and reduced nutrient availability which favor the accumulation of damage products and cell death [52]. Apoptotic, necrotic/necroptotic, and autophagic cells within the tumor microenvironment release damage-associated molecular patterns (DAMPs) which represent mediators including S100 proteins, ATP, heat shock proteins, hyaluronan, HMGB1 (high mobility group box 1), and calcireticulin [53] and are sensed by pattern recognition receptors (PRRs) such as toll-like receptors [54]. Such damage products in the presence of an acidified and hypoxic microenvironment can act as fusogenic triggers for aberrant spontaneous cell fusion or a so-called process of “accidental cell fusion” [55]. Previous work has demonstrated that hypoxia-induced apoptosis stimulates fusion between MSCs and breast tumor cells and generated hybrids accompanied by enhanced migratory capacity [56]. Peptides and proteins as well as small molecular metabolites and ions can destabilize the lipid bilayer of adjacent cell membranes and thereby contribute to spontaneous cell fusion by different mechanisms [57]. Similar effects of accidental cell fusion appear to be involved during spontaneous formation of hybrid cells following close interaction of MDA-MB-231 cells with MSC. The appearance of yellow fluorescing cells during co-culture of eGFP-transduced MSC with mcherry-transduced breast cancer cells suggested hybrid cell formation by cell fusion. Further confirmation was obtained by synchronous expression of both fluorescence genes at least in MDA-hyb2 cells. Moreover, MDA-hyb1 and MDA-hyb2 cells display a pseudo-triploid karyotype similar to MDA-MB-231 cells and a male phenotype acquired from MSC thus representing a combined genome from both parental cell types. Together, these effects support a mechanism via cell fusion rather than hybrid formation via entosis which includes degradation of the target cell genome. Fusion-driven hybrid cell formation combines the genomic parts from both parental donors by a hetero-to-synkaryon transition during subsequent cell division [58].

Although this breast cancer cell / MSC fusion process itself represents a rare event, only few of the resulting hybrid cells are capable of cell cycle progression due to regulatory impairments and replicative stress of the two different nuclei. Following initial aneuploidy and chromosomal instability of proliferative active hybrid cells, however, an immediate post-fusion selection process developed different genetically stable pseudo-triploid clones including MDA-hyb1 and MDA-hyb2 populations. Similar findings of rapid genomic post-fusion stabilization of hybrid cells without continuing prominent genetic and phenotypic plasticity were also obtained following PEG-induced xenogenic fusion of the small intestinal rat epithelial crypt cell line IEC-6 with human HeLa cervical carcinoma cells [59]. Spontaneous fusion of human bone marrow–derived multipotent stromal cells with breast carcinoma cell lines demonstrated mixed gene expression profiles and increased DNA ploidy [60]. Moreover, cancer cells can undergo homofusion to generate highly tumorigenic polyploid giant cancer cells. Previous work reported spontaneous fusion of the eGFP-Neo-transfected M13SV1 breast epithelial cell line with MDA-MB-231-Hyg [61] and with MDA-MB-435-Hyg breast cancer cells [62] which was accompanied by increased migratory activity and malignancy. Likewise, in vitro proliferative capacity and prominent telomerase activity paralleled by markedly elevated expression levels of genes associated with migration, EMT, and metastatic behavior were detectable in MDA-hyb1 and MDA-hyb2 hybrids in contrast to the parental MDA-MB-231 cells. Our data were further substantiated in vivo by the significantly enhanced MDA-hyb1- and MDA-hyb2-induced tumor growth and the rapid formation of organ metastases suggesting substantial increase in breast cancer metastatic capacity and malignancy of these hybrid cells. These findings are also in line with previous concepts suggesting that cancer cell fusion represents a potential mechanism of tumor metastases [63]. However, chemotherapeutic sensitivity was elevated in the hybrid populations compared to the parental MDA-MB-231 breast cancer cells.

Differences in malignancy are observed by correlating the properties of the two hybrid populations themselves. Thus, MDA-hyb1 presented higher in vitro proliferation as compared to MDA-hyb2 and accordingly, a faster tumor growth in vivo associated with a larger network of distant organ metastases which may be related in part to elevated S100A4 and more MDA-MB-231-like properties in MDA-hyb1. Together with the characteristics of the hybrid cells these data indicated that MDA-hyb2 cells carry more MSC-like properties and express a less aggressive tumor phenotype than MDA-hyb1.

Although previous work suggested that tumor-associated aberrant MSC contribute to tumor cell protection either directly by expression of protective extracellular matrix proteins and/or indirectly by promoting a carcinoma stem cell niche [41, 64, 65], the involvement of MSC in MDA-hyb1 and MDA-hyb2 cell formation displays increased sensitivity to various chemotherapeutic compounds.

## Conclusions

Altogether, tumor cell fusion by breast cancer/MSC hybrid cell formation enhanced the diversity of the tumor, accelerated tumor growth, increased metastases formation, and progressively altered the chemotherapeutic responsiveness whereby hybrid cells exhibited tumorigenic differences according to the acquisition of certain parental cell characteristics and properties.

## Additional files

Additional file 1: Figure S9. RNA microarray analysis. Gene expression data file for comparison between parental MSC and hybrid cells as well as between corresponding cancer and hybrid cells. Data are also stored at the NCBI-GEO database with the accession no. #GSE100551. (XLS 3897 kb)

Additional file 2: Figure S1. Isolation of MDA-hyb1 and MDA-hyb2 cells. Co-culture was performed between human MSC051212^GFP^ P3 20d and MDA-MB-231^cherry^ breast cancer cells for 6 days in MSC medium (cell ratio 60:40) resulted in the appearance of yellow-colored hybrid cells. These hybrid cells were separated for double-labeled (mcherry and eGFP) cells in two steps by repeated fluorescence-activated cell sorting (FACS). Hybrid cells were collected in microtiter plates with one to two hybrid cells/well and subsequent cell cloning. (PDF 252 kb)

Additional file 3: Figure S2. Karyotype analysis of MDA-hyb1 and MDA-hyb2 cells. Following preparation of metaphase chromosomes by colchicine treatment and Giemsa staining karyotype analysis was performed in MSC051212^GFP^ and MDA-MB-231^cherry^ cells as compared to MDA-hyb1 and MDA-hyb2 cells. (PDF 315 kb)

Additional file 4: Figure S3. Cell cycle analysis of MDA-hyb1 and MDA-hyb2 cells. Cell cycle analysis was performed by DNA labeling and subsequent FACS measurements in steady state MSC^GFP^ and MDA-MB-231^cherry^ cells as compared to MDA-hyb1 and MDA-hyb2 cells. The cell cycle shift of MDA-MB-231^cherry^, MDA-hyb1, and MDA-hyb2 cells towards increased fluorescence intensities as compared to MSC^GFP^demonstrated an increased amount of DNA and accordingly, aneuploidy in these three cell populations in contrast to a normal diploid set of chromosomes in MSC^GFP^. Quantification of cell cycle phases was performed using FlowJo software. (PDF 192 kb)

Additional file 5: Figure S4. Ki67 expression in MDA-hyb1 and MDA-hyb2 cells. Cell cultures of MDA-MB-231^cherry^, MDA-hyb1 and MDA-hyb2 cells were fixed and stained with Ki67 (upper panel). Quantification was performed by cell counting of four independent specimen and calculated as percentage of Ki67-positive cells. Data represent the mean + s.d. (*n* = 4). Expression of Ki67 was performed in MSC051212^GFP^, MDA-MB-231^cherry^, MDA-hyb1, and MDA-hyb2 cells by RT-PCR analysis (lower panel). Unaltered mRNA levels of GAPDH served as a control. (PDF 368 kb)

Additional file 6: Figure S5. MSC characteristic markers. Relative expression analysis based on the RNA microarray data of some characteristic mesenchymal stem-like markers was calculated for MDA-MB-231 cells and the hybrid populations MDA-hyb1 and MDA-hyb2. For relative evaluations the expression levels of MSC were used as a control (set to 100%). (PDF 175 kb)

Additional file 7: Figure S6. Analysis of disease and function genes. Relative dominance and importance of certain disease- and function-associated gene clusters in hybrid cells and the parental MDA-MB-231 and MSC051212 were calculated as –log(*p*-values). Evaluation was performed by relative expression levels of these disease- and function-associated genes in MDA-hyb1 cells in relationship to both parental MDA-MB-231 and MSC051212, respectively, and in MDA-hyb2 cells in relationship to both parental MDA-MB-231 and MSC051212, respectively (left panel). In further summarizing disease- and function-associated clusters obtained from Ingenuity pathway analysis, the relationship of MDA-hyb1 to MDA-MB-231 cells (right upper panel) and the relationship of MDA-hyb2 to MDA-MB-231 cells (right lower panel) are presented. (PDF 501 kb)

Additional file 8: Figure S7. Transfection efficiency. Transfection efficiency for the siRNA knock-down experiments was evaluated following transfection of MDA-MB-231 cells with 25 nM of the green fluorescing siGLO^green^ control vector. (PDF 172 kb)

Additional file 9: Figure S8. Chemotherapeutic responsiveness of MDA-hyb1 and MDA-hyb2 cells. Compared to the parental MDA-MB-231 cells, MDA-hyb1 and MDA-hyb2 cells were treated with 1 μM of the chemotherapeutic compounds taxol, cisplatin, methotrexate (MTX), epirubicin, and foretinib for 24 h up to 72 h, respectively. Relative fluorescence was evaluated by fluoroskan assay representing the mean ± s.d. (*n* = 10). (PDF 96 kb)

## Abbreviations

ASCT-2: Alanine serine and cysteine selective transporter-2
ATP: Adenosine triphosphate
CCL: Chemokine (C-C motif) ligand
CCR5: C-C chemokine receptor type 5
CSC: Cancer stem-like cells
CXCL: Chemokine (C-X-C motif) ligand
CXCR4: C-X-C chemokine receptor type 4
EGF: Epidermal growth factor
eGFP: Enhanced green fluorescent protein
EMT: Epithelial-mesenchymal transition
FACS: Fluorescence-activated cell sorting
FCS: Fetal calf serum
FGF: Fibroblast growth factor
HMGB1: High mobility group box 1
ICAM-1: Intercellular adhesion molecule 1
MFSD-2A: Major facilitator superfamily domain containing 2A
MSC: Mesenchymal stroma/stem cells
MUC-1: Mucin-1
PDGF: Platelet-derived growth factor
PRR: Pattern recognition receptor
RT-PCR: Reverse transcription polymerase chain reaction
SDF-1: Stromal cell-derived factor-1
siRNA: Small-interfering RNA
STR: Short tandem repeat
TGF-β: Transforming growth factor beta
TRAP: Telomeric repeat amplification protocol
