# Supplementary material for: Enhanced metastatic capacity of breast cancer cells after interaction and hybrid formation with mesenchymal stroma/stem cells (MSC)
Source: Cell Commun Signal. 2018 Jan 5;16:2. doi: 10.1186/s12964-018-0215-4 (PMC5795285; doi:10.1186/s12964-018-0215-4)

suppl. Fig.S1

# Isolation of MDA-MSC hybrid cells

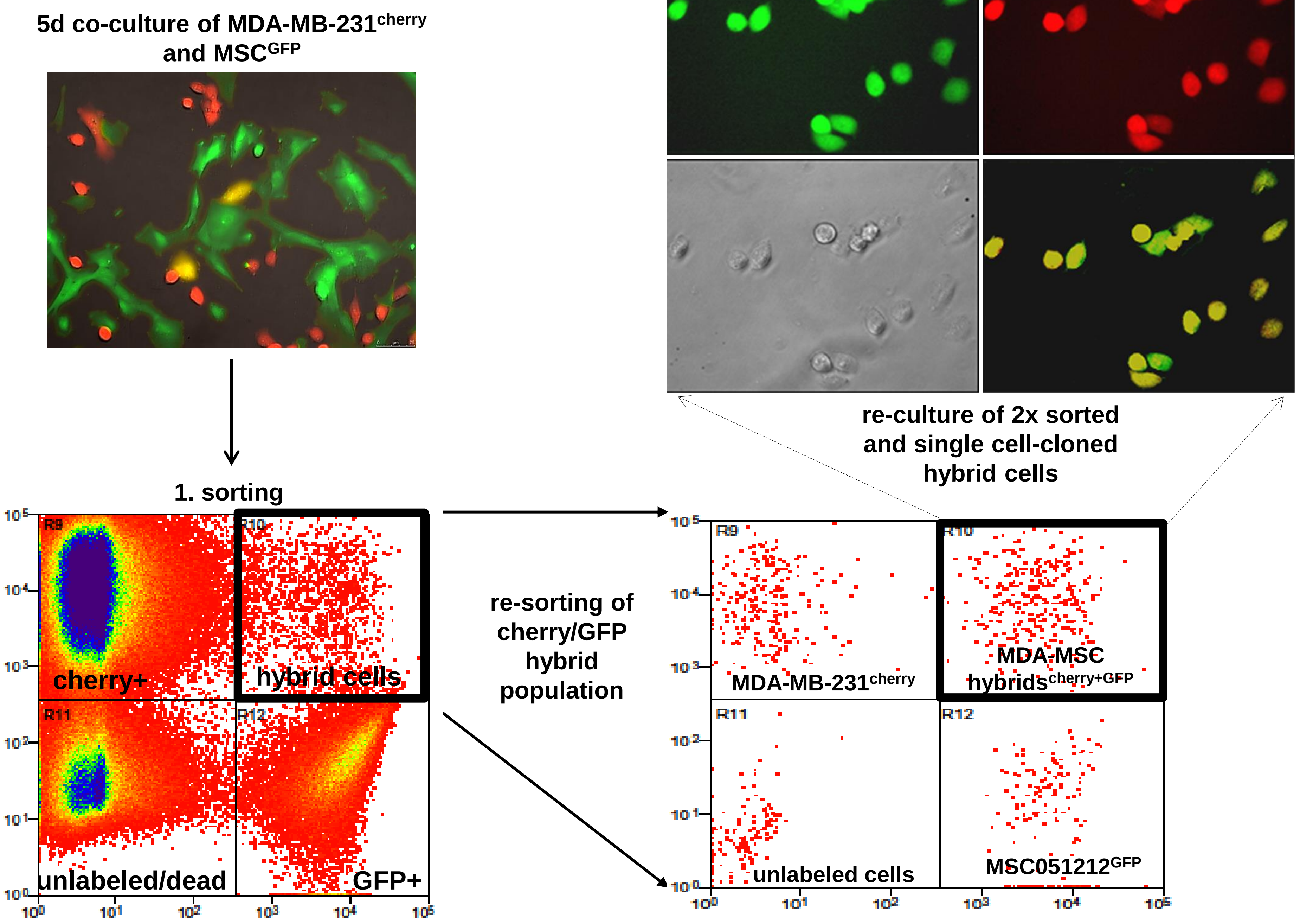

Supplement: Supplementary file 2 — Isolation of MDA-hyb1 and MDA-hyb2 cells. Co-culture was performed between human MSC051212GFP P3 20d and MDA-MB-231cherry breast cancer cells for 6 days in MSC medium (cell ratio 60:40) resulted in the appearance of yellow-colored hybrid cells. These hybrid cells were separated for double-labeled (mcherry and eGFP) cells in two steps by repeated fluorescence-activated cell sorting (FACS). Hybrid cells were collected in microtiter plates with one to two hybrid cells/well and subsequent cell cloning. (PDF 252 kb) [file 12964_2018_215_MOESM2_ESM.pdf]
