# Supplementary figures and images for: Enhanced metastatic capacity of breast cancer cells after interaction and hybrid formation with mesenchymal stroma/stem cells (MSC)
Source: Cell Commun Signal. 2018 Jan 5;16:2. doi: 10.1186/s12964-018-0215-4 (PMC5795285; doi:10.1186/s12964-018-0215-4)

# karyotyping

MDA-MB-231<sup>cherry</sup>

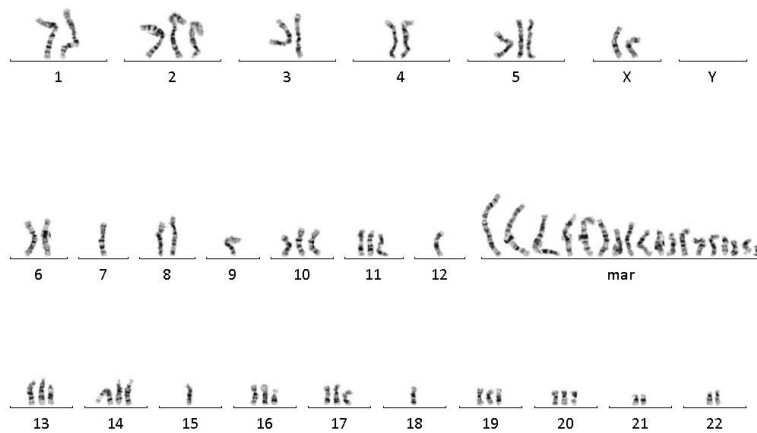

MSC051212<sup>GFP</sup>

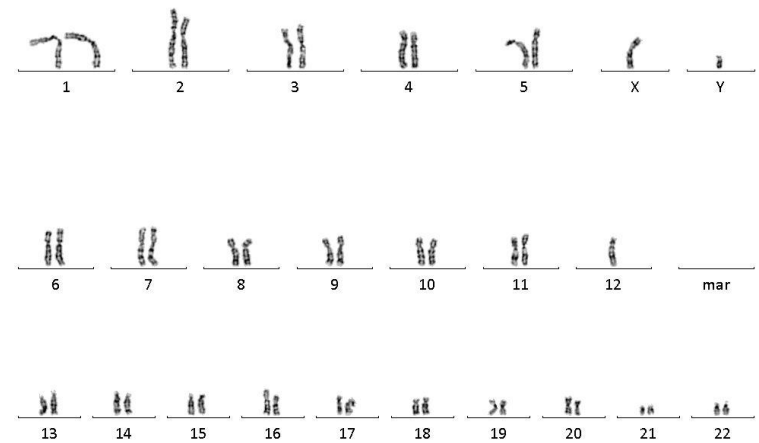

MDA-hyb1

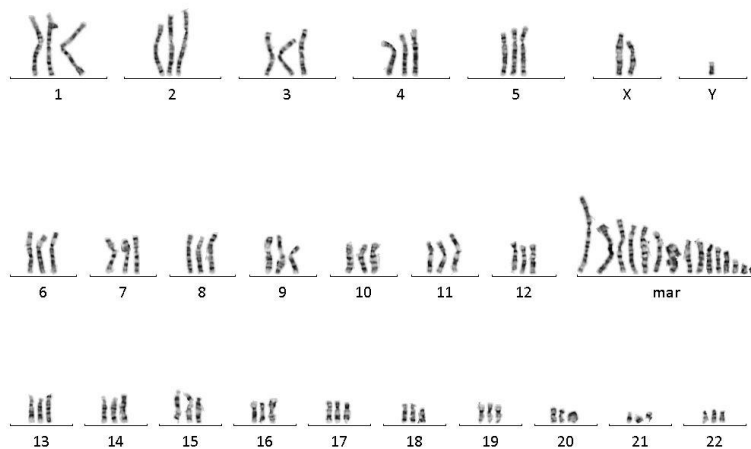

MDA-hyb2

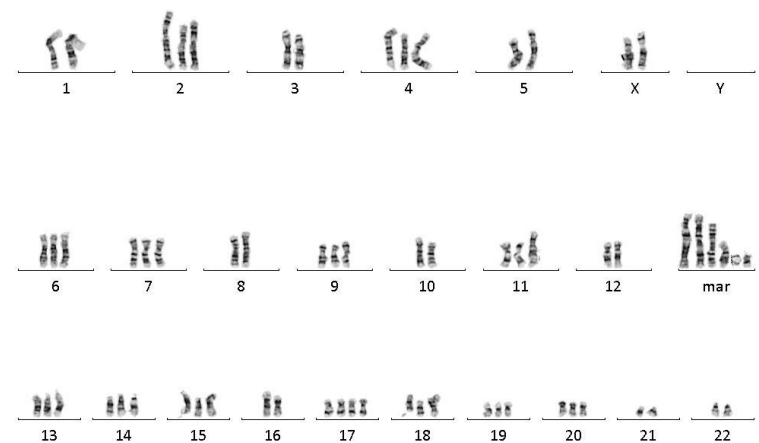

Supplement: Supplementary file 3 — Karyotype analysis of MDA-hyb1 and MDA-hyb2 cells. Following preparation of metaphase chromosomes by colchicine treatment and Giemsa staining karyotype analysis was performed in MSC051212GFP and MDA-MB-231cherry cells as compared to MDA-hyb1 and MDA-hyb2 cells. (PDF 315 kb) [file 12964_2018_215_MOESM3_ESM.pdf]

# cell cycle analysis

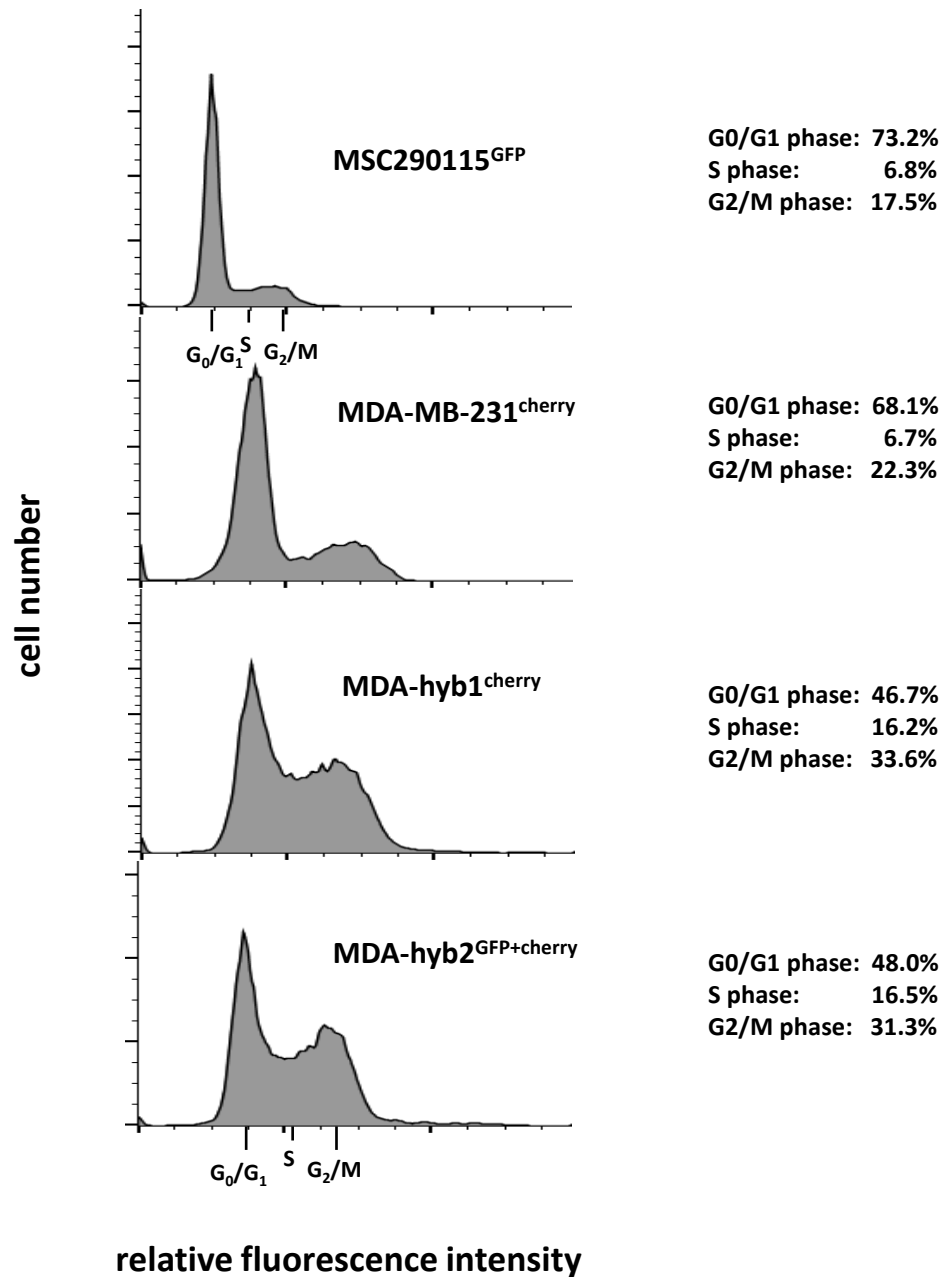

Supplement: Supplementary file 4 — Cell cycle analysis of MDA-hyb1 and MDA-hyb2 cells. Cell cycle analysis was performed by DNA labeling and subsequent FACS measurements in steady state MSCGFP and MDA-MB-231cherry cells as compared to MDA-hyb1 and MDA-hyb2 cells. The cell cycle shift of MDA-MB-231cherry, MDA-hyb1, and MDA-hyb2 cells towards increased fluorescence intensities as compared to MSCGFPdemonstrated an increased amount of DNA and accordingly, aneuploidy in these three cell populations in contrast to a normal diploid set of chromosomes in MSCGFP. Quantification of cell cycle phases was performed using FlowJo software. (PDF 192 kb) [file 12964_2018_215_MOESM4_ESM.pdf]

## Ki67 staining

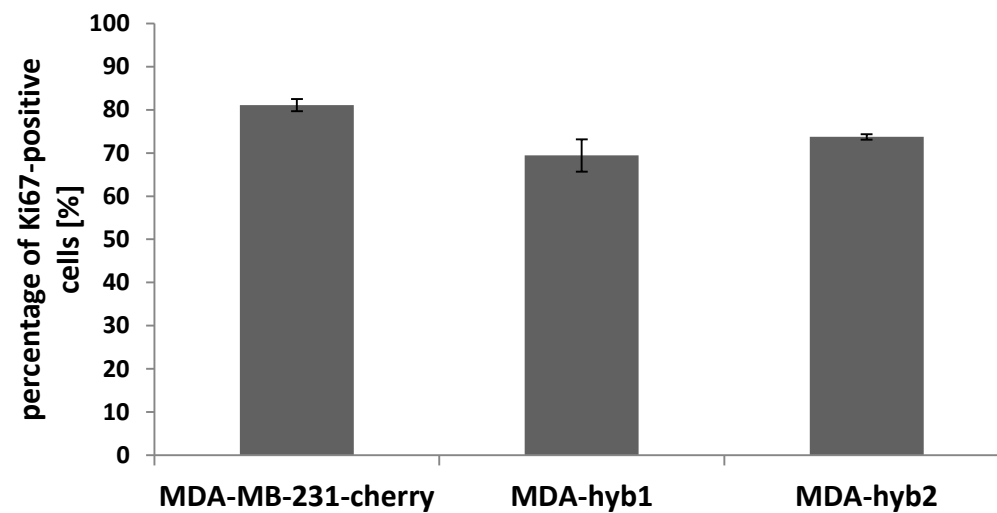

## Ki67 PCR

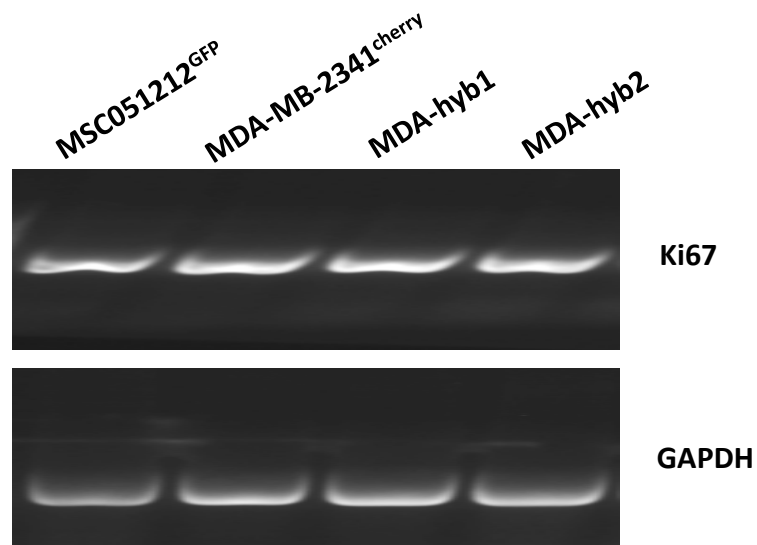

Supplement: Supplementary file 5 — Ki67 expression in MDA-hyb1 and MDA-hyb2 cells. Cell cultures of MDA-MB-231cherry, MDA-hyb1 and MDA-hyb2 cells were fixed and stained with Ki67 (upper panel). Quantification was performed by cell counting of four independent specimen and calculated as percentage of Ki67-positive cells. Data represent the mean + s.d. (n = 4). Expression of Ki67 was performed in MSC051212GFP, MDA-MB-231cherry, MDA-hyb1, and MDA-hyb2 cells by RT-PCR analysis (lower panel). Unaltered mRNA levels of GAPDH served as a control. (PDF 368 kb) [file 12964_2018_215_MOESM5_ESM.pdf]

## MSC stemness marker

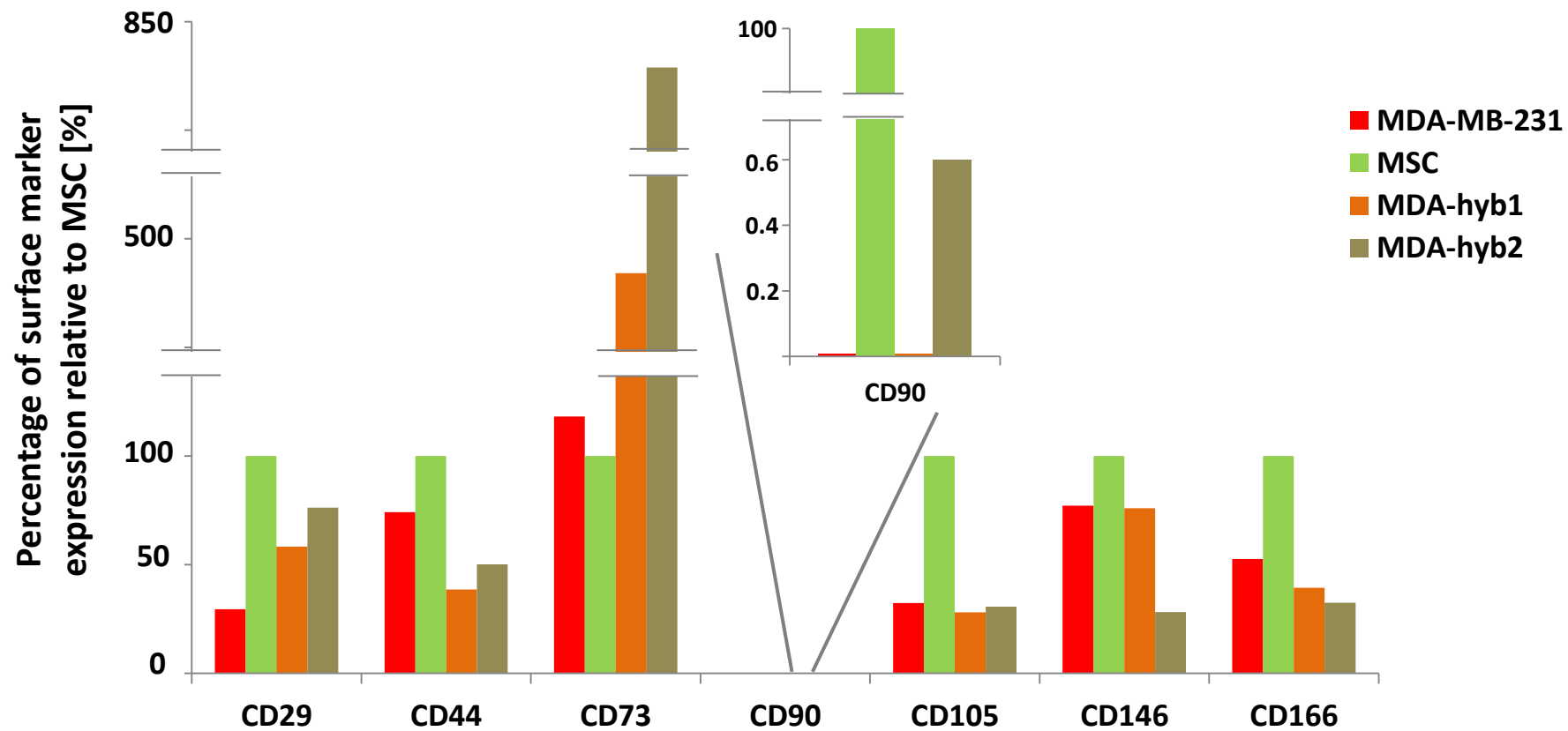

Supplement: Supplementary file 6 — MSC characteristic markers. Relative expression analysis based on the RNA microarray data of some characteristic mesenchymal stem-like markers was calculated for MDA-MB-231 cells and the hybrid populations MDA-hyb1 and MDA-hyb2. For relative evaluations the expression levels of MSC were used as a control (set to 100%). (PDF 175 kb) [file 12964_2018_215_MOESM6_ESM.pdf]

MDA-MB-231 control cells

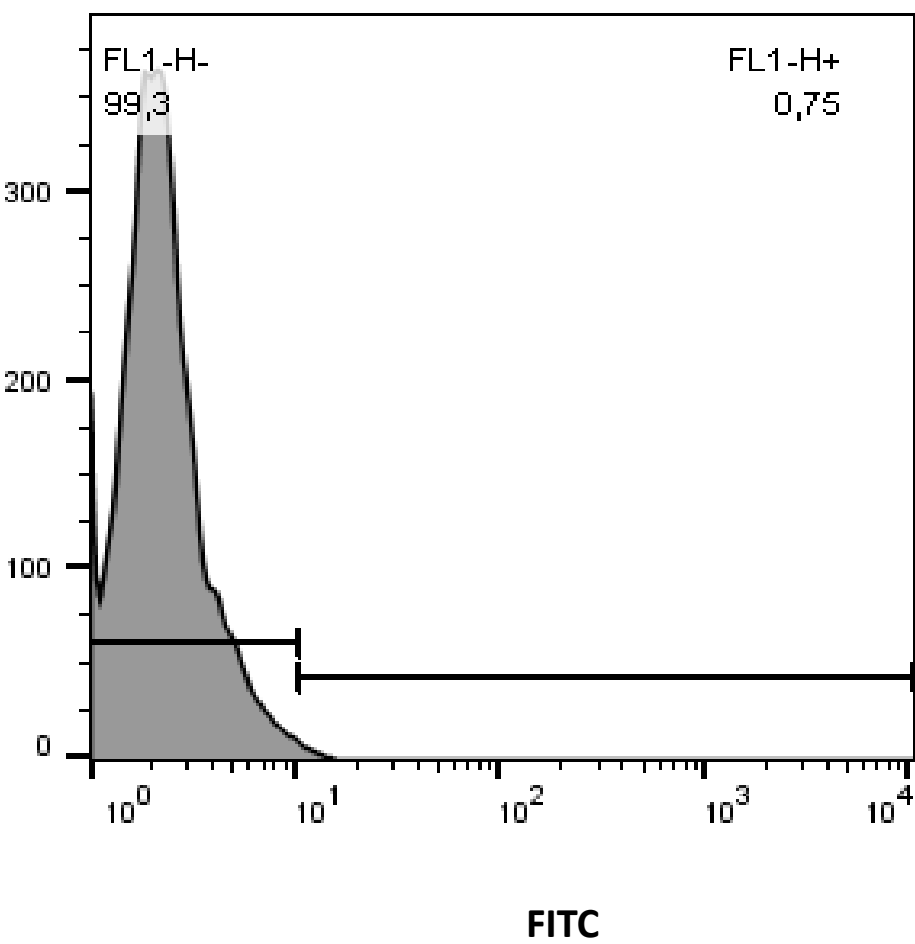

siGLO<sup>Green</sup> transfected  
MDA-MB-231 cells

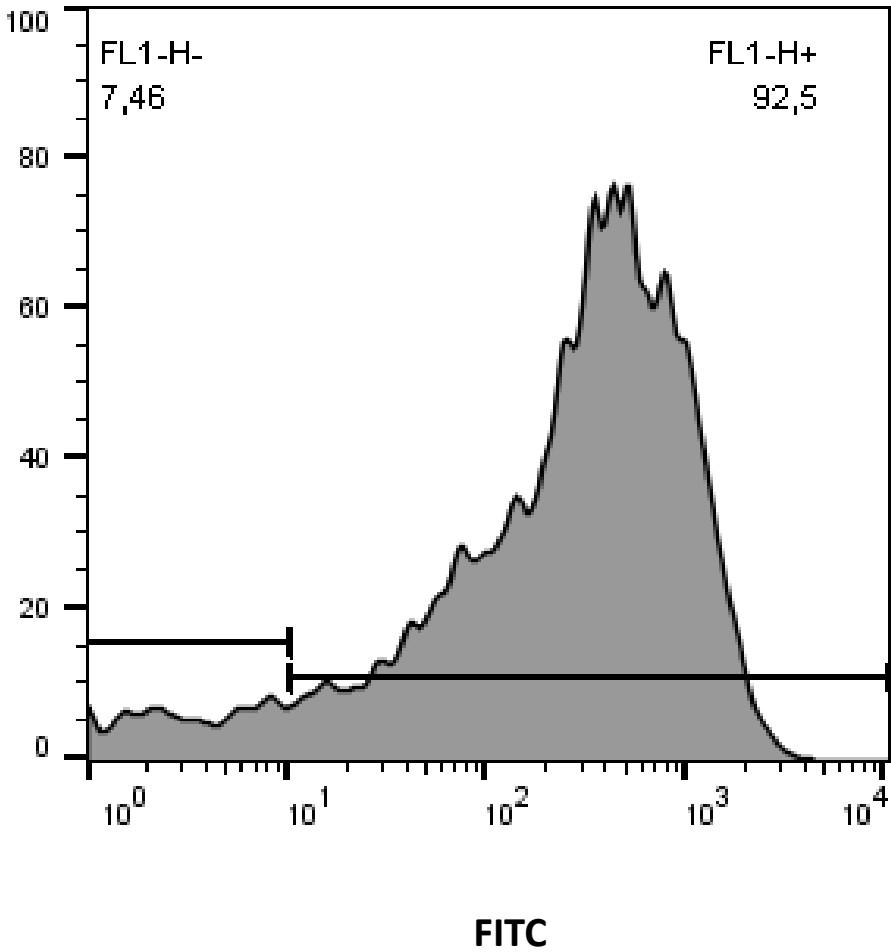

Supplement: Supplementary file 8 — Transfection efficiency. Transfection efficiency for the siRNA knock-down experiments was evaluated following transfection of MDA-MB-231 cells with 25 nM of the green fluorescing siGLOgreen control vector. (PDF 172 kb) [file 12964_2018_215_MOESM8_ESM.pdf]

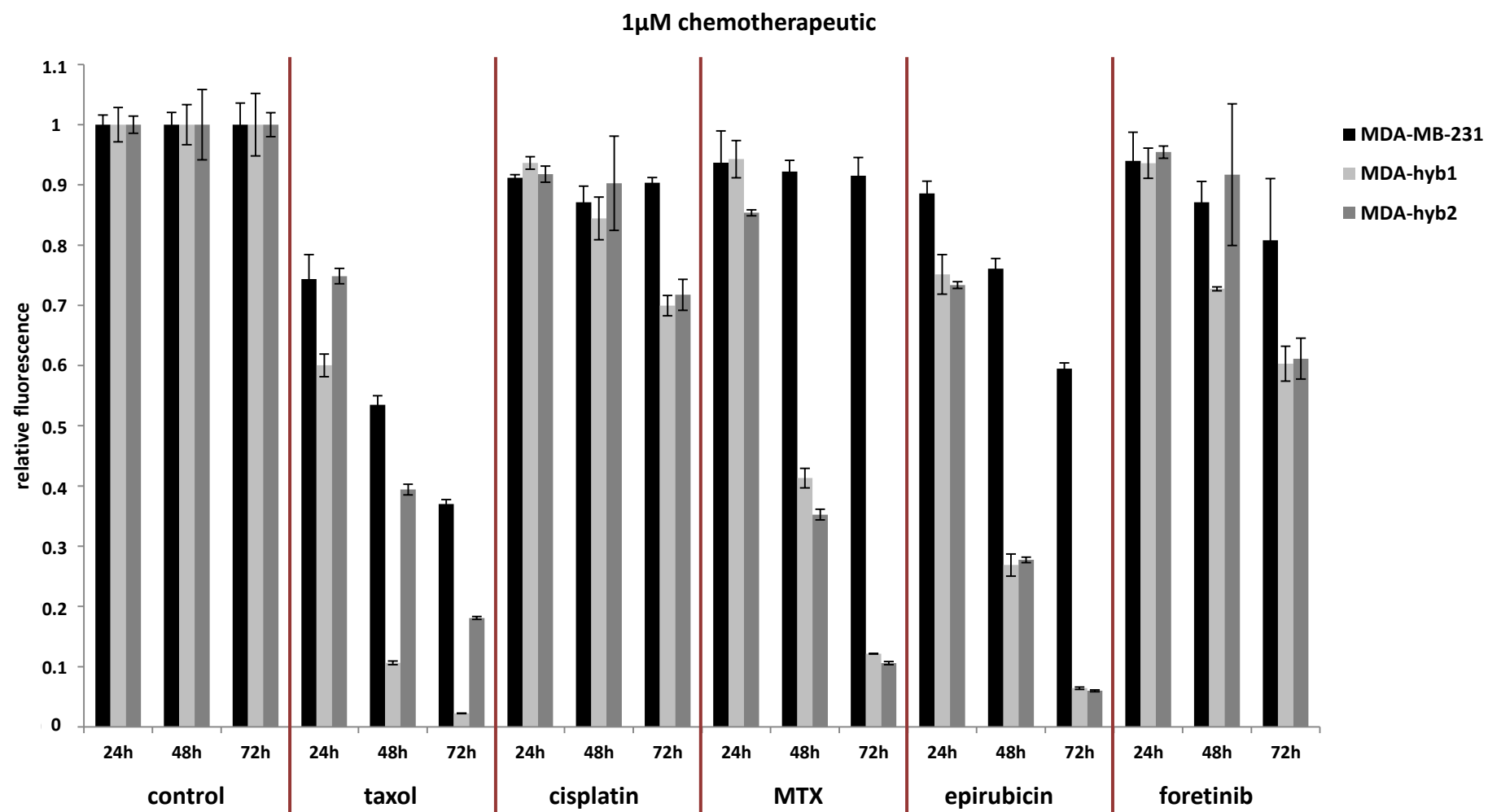

Supplement: Supplementary file 9 — Chemotherapeutic responsiveness of MDA-hyb1 and MDA-hyb2 cells. Compared to the parental MDA-MB-231 cells, MDA-hyb1 and MDA-hyb2 cells were treated with 1 μM of the chemotherapeutic compounds taxol, cisplatin, methotrexate (MTX), epirubicin, and foretinib for 24 h up to 72 h, respectively. Relative fluorescence was evaluated by fluoroskan assay representing the mean ± s.d. (n = 10). (PDF 96 kb) [file 12964_2018_215_MOESM9_ESM.pdf]
