# Supplementary material for: Enhanced metastatic capacity of breast cancer cells after interaction and hybrid formation with mesenchymal stroma/stem cells (MSC)
Source: Cell Commun Signal. 2018 Jan 5;16:2. doi: 10.1186/s12964-018-0215-4 (PMC5795285; doi:10.1186/s12964-018-0215-4)

suppl. Fig.S6

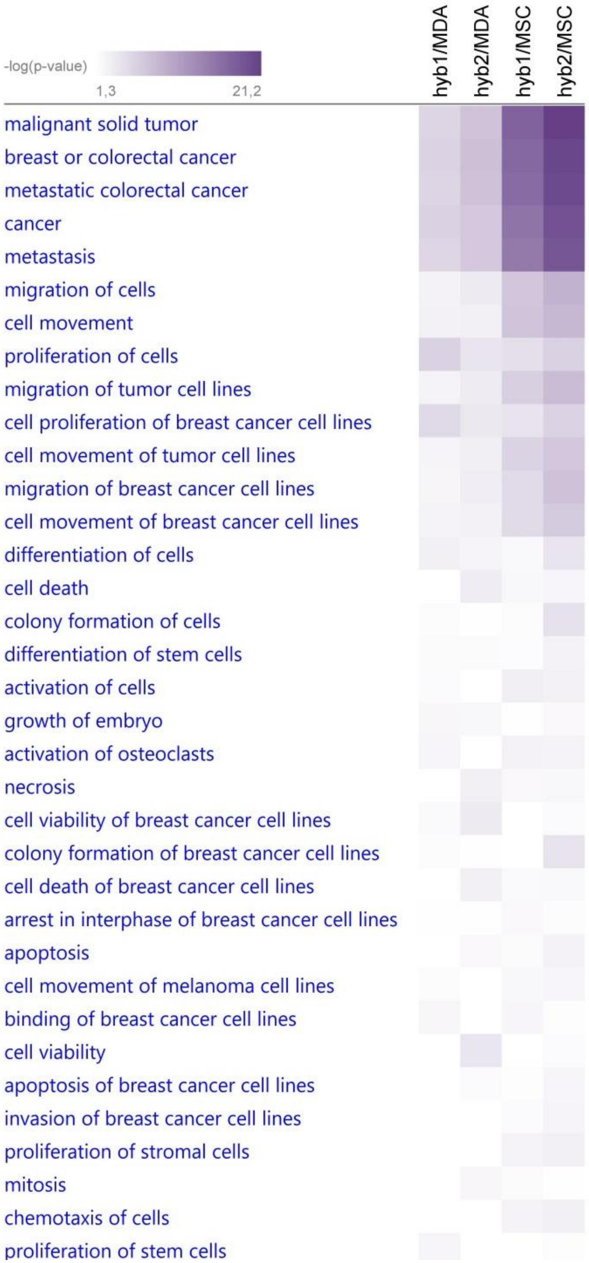

Diseases and functions

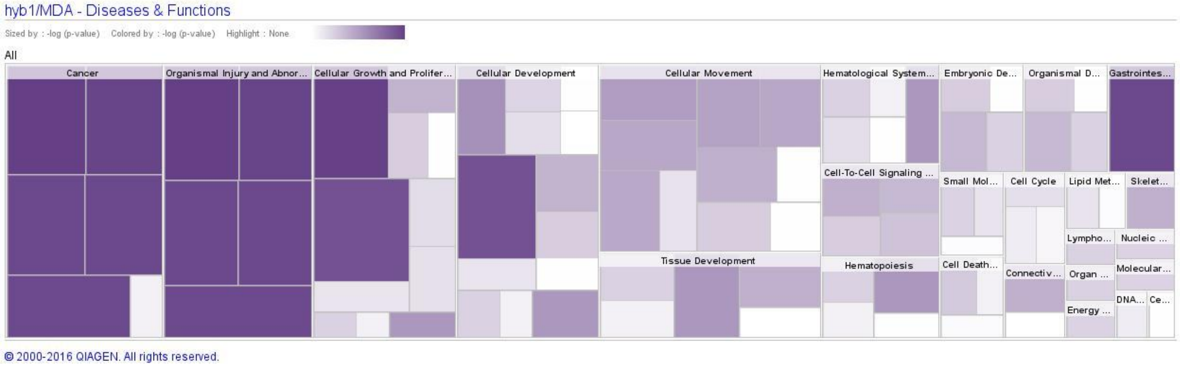

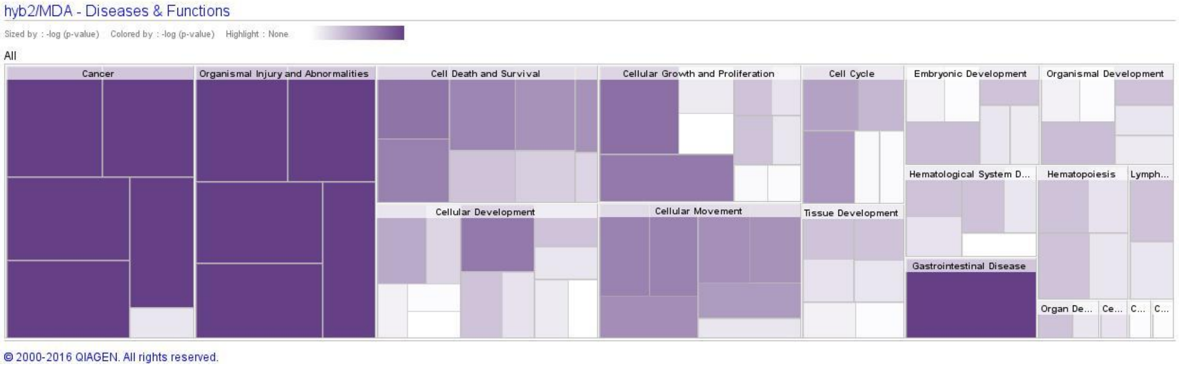

Supplement: Supplementary file 7 — Analysis of disease and function genes. Relative dominance and importance of certain disease- and function-associated gene clusters in hybrid cells and the parental MDA-MB-231 and MSC051212 were calculated as –log(p-values). Evaluation was performed by relative expression levels of these disease- and function-associated genes in MDA-hyb1 cells in relationship to both parental MDA-MB-231 and MSC051212, respectively, and in MDA-hyb2 cells in relationship to both parental MDA-MB-231 and MSC051212, respectively (left panel). In further summarizing disease- and function-associated clusters obtained from Ingenuity pathway analysis, the relationship of MDA-hyb1 to MDA-MB-231 cells (right upper panel) and the relationship of MDA-hyb2 to MDA-MB-231 cells (right lower panel) are presented. (PDF 501 kb) [file 12964_2018_215_MOESM7_ESM.pdf]
